# The influence of exercise identity on college students' perceived social self-efficacy: chain mediating effect test

**DOI:** 10.3389/fpsyg.2025.1751057

**Published:** 2026-01-29

**Authors:** Wenhua Jin, Bin Li, Xiang Zhao

**Affiliations:** 1 School of Physical Education, Ningxia University, Ningxia, China; 2 School of Physical Education, Huaibei Normal University, Huaibei, China

**Keywords:** college students, emotion regulation, exercise identity, motivation for physical activity, perceived social self-efficacy

## Abstract

**Objective:**

This study aimed to examine the influence of exercise identity on college student's perceived social self-efficacy (PSSE), and to explore the chain-mediating roles of emotion regulation and motivation for physical activity in this relationship.

**Methodology:**

A total of 1,029 Chinese college students (mean age = 18.9 years, SD = 0.80) completed standardized measures, including the Exercise Identity Scale (EIS), Perceived Social Self-Efficacy Scale (PSSE), Difficulties in Emotion Regulation Scale (DERS-16), and Motivation for Physical Activity Measure-Revised (MPAM-R). Data were analyzed using descriptive statistics, correlation analysis, and mediation analysis (PROCESS Model 6 with 5,000 bootstrap samples), controlling for gender and age.

**Results:**

Exercise identity showed a significant positive direct effect on PSSE (β = 0.443, *p* < 0.001). Emotion regulation and physical activity motivation each served as independent mediators, and also functioned sequentially in a chain-mediation pathway. Specifically, motivation alone accounted for 37.25% of the total effect, emotion regulation alone for 7.45%, and the sequential path (Exercise Identity → Emotion Regulation → Motivation → PSSE) explained an additional 14.88%. The results collectively supported a significant chain mediation model.

**Conclusion:**

A stronger exercise identity is associated with higher perceived social self-efficacy among college students, both directly and through the mediating roles of emotion regulation and autonomous motivation for physical activity. Emotion regulation facilitates higher-quality motivation, which in turn enhances social confidence, forming a positive psychological pathway from exercise identity to social adaptation. These findings underscore the value of promoting exercise identity and emotion regulation skills to support college student's psychosocial wellbeing and adaptive functioning.

## 1 Introduction

The university period represents a crucial developmental stage in emerging adulthood, characterized by profound cognitive, emotional, and social transformations (Han, 2022). During this time, individuals face the dual challenges of consolidating a coherent self-identity and adapting to increasingly complex interpersonal environments (Wei et al., 2025). Perceived Social Self-Efficacy (PSSE): defined as one's belief in the ability to initiate and maintain social relationships, manage social interactions, and gain peer acceptance (Smith and Betz, 2000), is an essential psychological resource during this transition. High levels of PSSE have been associated with greater psychological wellbeing, stronger social connectedness, and reduced anxiety (Wang et al., 2015), whereas low PSSE predicts social withdrawal, loneliness, and maladjustment (Tang and Guo, 2021). Understanding how PSSE can be strengthened among college students therefore carries both theoretical and practical significance.

Beyond its well-established physical benefits, exercise is increasingly recognized as a catalyst for psychological growth and social development (Biddle and Asare, 2011). Within this domain, exercise identity, defined as the extent to which an individual identifies with the “exerciser” role as a component of their self-concept (Anderson and Cychosz, 1994), has garnered growing research attention. Individuals with a strong exercise identity tend to engage more consistently in physical activity and experience positive psychological outcomes such as enhanced self-esteem and resilience (Porter et al., 2024). Yet, the mechanisms through which exercise identity influences social cognitive constructs such as PSSE remain underexplored. It is plausible that this influence occurs indirectly, through emotion-related and motivational processes that mediate the relationship between self-concept and social confidence. Accordingly, this study proposes and empirically tests a chain mediation model, in which emotion regulation and motivation for physical activity sequentially mediate the association between exercise identity and PSSE.

### 1.1 The relationship between exercise identity and perceived social self-efficacy

According to identity theory, the self is composed of multiple interrelated role identities that guide behavior through internalized expectations and self-standards (Stets and Carter, 2012). Each identity provides a framework for interpreting experiences and regulating behavior in ways that maintain congruence between the self-concept and social roles (Stets and Burke, 2000). When individuals internalize the exerciser role as a salient part of their self-definition, they are motivated to enact behaviors that affirm and express this identity (Vlachopoulos et al., 2011). These behaviors often include consistent participation in physical activity contexts such as fitness classes, sports teams, and gym environments, settings that naturally involve social cooperation, communication, and mutual support (Reifsteck et al., 2016). Notably, physical activity participation also encompasses individual forms (e.g., running, cycling, home workouts), which equally contribute to the development of exercise identity and subsequent social self-efficacy through distinct mechanisms.

Through repeated engagement in these socially interactive exercise experiences (for group exercisers) or self-directed physical activity (for individual exercisers), individuals accumulate social mastery experiences, a core source of self-efficacy as conceptualized by Bandura (1997). For group exercisers, successfully coordinating with teammates, Successfully coordinating with teammates, achieving shared goals, or resolving minor interpersonal challenges within these contexts fosters a sense of competence and confidence in one's ability to manage social situations (Ma et al., 2023). For individual exercisers, consistent self-discipline, goal-setting, and self-monitoring during solo activity enhance self-regulation and perceived personal efficacy, which generalize to social domains by boosting self-assurance in initiating and maintaining social interactions (Gu, 2025). Over time, these domain-specific successes generalize to broader social domains, contributing to higher perceived social self-efficacy (PSSE) in academic, peer, and community interactions (Brouwer et al., 2010).

Furthermore, the social validation that accompanies exercise participation—whether through peer recognition in group settings or social approval of an active lifestyle in individual contexts, reinforces both identity salience and social confidence (Tucker et al., 2023). Even individual exercisers often experience indirect social benefits, such as increased approachability, shared identity with other active individuals in casual social encounters, or enhanced self-presentation confidence derived from physical fitness (Haslam et al., 2016). In this way, the exercise identity not only motivates behavioral consistency but also serves as a psychological bridge linking physical engagement with social functioning (Jelalian et al., 2011). Empirical studies have consistently demonstrated that regular physical activity predicts greater social competence, peer connectedness, and overall self-efficacy (Dishman et al., 2008), suggesting that identity-driven exercise engagement plays a crucial role in psychosocial development.

Taken together, these theoretical and empirical considerations support the expectation that individuals with a stronger exercise identity will exhibit greater confidence in their ability to initiate and maintain effective social interactions. Therefore, Hypothesis 1 (H1) was proposed: Stronger exercise identity will positively predict higher levels of perceived social self-efficacy among college students.

### 1.2 The mediating role of emotion regulation

Emotion regulation, defined as the process of monitoring, evaluating, and modifying emotional reactions, is a cornerstone of adaptive psychological functioning and social adjustment (Phillips and Power, 2007). It enables individuals to maintain emotional stability and respond constructively to internal and external challenges (Li and Guo, 2025). Within the domain of physical activity, emotion regulation is not only an outcome but also an essential psychological mechanism through which exercise exerts its broader benefits (Zhang et al., 2025).

Individuals with strong exercise identities are more likely to engage consistently in physical activity, which enhances emotional control and resilience to stress (Gosadi, 2024). Regular exercise provides structured opportunities for individuals to experience and manage varying emotional states, such as motivation, frustration, and accomplishment, within a supportive and goal-directed environment (Zhou, 2024). Through these repeated emotional experiences, exercisers gradually develop the ability to recognize, interpret, and modulate their emotions more effectively (Tian et al., 2025). At the neurobiological level, exercise contributes to the down-regulation of negative affect and the promotion of positive mood states through mechanisms such as endorphin release and improved autonomic balance (Guo and Zhang, 2022). Over time, these physiological and psychological adaptations foster a stable emotional profile and higher emotional competence (Wang and Xu, 2025).

Emotion regulation is closely intertwined with social functioning. Individuals capable of effectively managing their emotions tend to be more adept at navigating complex social situations, demonstrating empathy, and sustaining positive interpersonal relationships (Tugade and Fredrickson, 2007). They can better manage conflict, recover from social stress, and project confidence in social interactions. In contrast, individuals with poor emotion regulation often experience elevated social anxiety, difficulties in emotional expression, and reduced social confidence (Lewis et al., 2006). From this perspective, emotion regulation serves as a psychological bridge connecting exercise identity to perceived social self-efficacy. By enhancing emotion regulation capacity through consistent exercise engagement, individuals strengthen their emotional stability and self-assurance, which in turn promote greater confidence and effectiveness in social interactions (Zhang et al., 2024). Therefore, Hypothesis 2 (H2) was proposed: Emotion regulation mediates the relationship between exercise identity and perceived social self-efficacy.

### 1.3 The mediating role of motivation for physical activity

According to Self-Determination Theory (SDT) (Deci and Ryan, 2012), motivation lies along a continuum ranging from controlled to autonomous regulation. Among these forms, intrinsic motivation-driven by enjoyment, curiosity, and personal satisfaction-produces the most enduring engagement and the strongest psychological benefits. When individuals internalize the exerciser role as part of their self-concept, they are more likely to behave in ways consistent with this identity, thereby reinforcing autonomous motivation (Hagger and Chatzisarantis, 2011). In this sense, a salient exercise identity not only reflects participation in physical activity but also functions as a motivational anchor that sustains long-term engagement aligned with one's self-view (Wilson et al., 2008).

The Motivation for Physical Activity Measure-Revised (MPAM-R) categorizes physical activity motivation into five domains: enjoyment, competence, appearance, fitness, and social interaction (Teixeira et al., 2012). Among these, enjoyment, competence, and social motives are particularly relevant to social self-efficacy, as they encourage individuals to participate in group-based or socially interactive exercise contexts. These environments provide repeated opportunities for cooperation, mutual feedback, and mastery experiences, all of which enhance perceived social competence and self-efficacy. Furthermore, intrinsic motivation has been found to enhance positive affect, psychological vitality, and overall self-confidence (Fortier et al., 2012). These affective and cognitive benefits extend beyond exercise settings to the social domain, where individuals with stronger intrinsic motivation display greater optimism, energy, and confidence in initiating and maintaining interpersonal relationships. The positive emotions and self-assurance generated through intrinsically motivated activity create a transfer effect, fostering stronger perceived social self-efficacy across different contexts.

In sum, motivation for physical activity serves as a critical psychological pathway through which exercise identity exerts its influence on social functioning. A strong exercise identity promotes self-determined motivation, which in turn enhances both the emotional and cognitive resources needed for successful social interaction.Therefore, Hypothesis 3 (H3) was proposed: Motivation for physical activity mediates the relationship between exercise identity and perceived social self-efficacy.

### 1.4 The chain mediating effect of emotion regulation and motivation for physical activity

Emotion regulation and motivation are functionally and conceptually interconnected psychological processes that jointly shape adaptive behavior. Effective emotion regulation enables individuals to manage affective responses and reinterpret exercise-related challenges as opportunities for growth rather than as sources of stress. This emotional adaptability facilitates the internalization of autonomous motivation, allowing individuals to perceive physical activity as inherently rewarding instead of externally imposed (Vega-Díaz et al., 2025). Conversely, when individuals are autonomously motivated, they are more likely to engage in behaviors that promote emotional balance and psychological wellbeing, further reinforcing their ability to regulate emotions effectively.

From the perspective of Self-Determination Theory (Deci and Ryan, 2012), emotion regulation supports the satisfaction of basic psychological needs-competence, autonomy, and relatedness-which in turn strengthen self-determined motivation for physical activity. This motivation not only sustains behavioral persistence but also enhances emotional vitality and confidence during social interactions (Fortier et al., 2012). Similarly, Identity Theory (Stets and Carter, 2012) posits that a well-defined self-concept, such as a strong exercise identity, organizes both emotional responses and motivational patterns in ways that reinforce role-consistent behaviors. Thus, individuals who identify strongly as exercisers are more likely to use adaptive emotion regulation strategies that cultivate intrinsic enjoyment, persistence, and social engagement within exercise contexts.

Collectively, these mechanisms suggest a chain-mediating pathway in which exercise identity first enhances emotion regulation ability, which subsequently promotes higher levels of autonomous motivation for physical activity. In turn, this increased intrinsic motivation fosters positive affective experiences, social participation, and interpersonal confidence, ultimately leading to greater perceived social self-efficacy (PSSE). The sequential process of Exercise Identity, Emotion Regulation, Motivation for Physical Activity, and PSS illustrates how the self-concept in the exercise domain exerts a cascading influence on social functioning through emotional and motivational systems. Therefore, Hypothesis 4 (H4) was proposed: Emotion regulation and motivation for physical activity exert a significant chain-mediating effect between exercise identity and perceived social self-efficacy, with the specific chain pathway being Exercise Identity → Emotion Regulation → Motivation for Physical Activity → Perceived Social Self-Efficacy.

**Figure 1:**
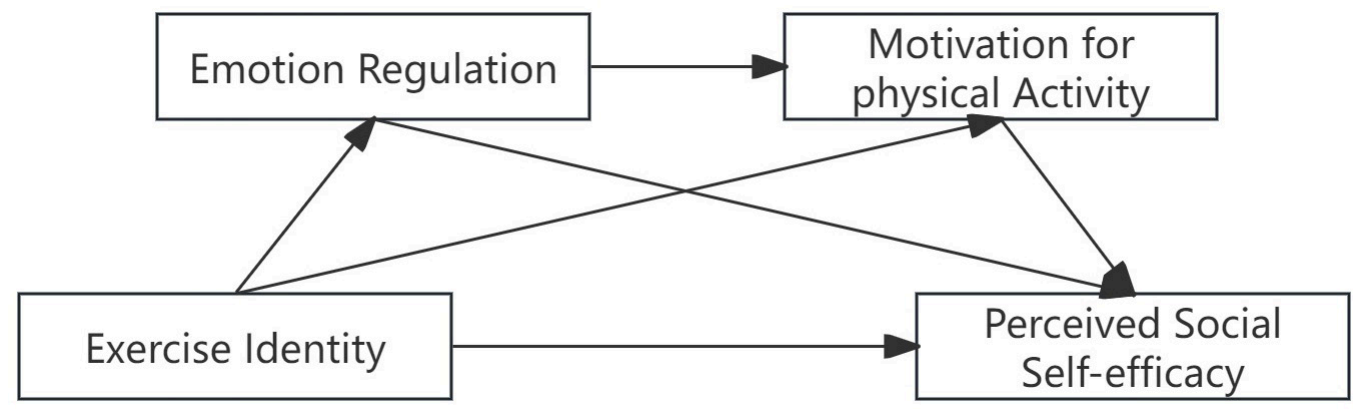
Conceptual model.

This study proposes and empirically validates a conceptual model elucidating how exercise identity shapes college students' perceived social self-efficacy through the intertwined mediating effects of emotion regulation and motivation for physical activity (Figure 1). As illustrated in the model, the core elements—exercise identity (predictor), emotion regulation and motivation for physical activity (sequential mediators), and perceived social self-efficacy (outcome)—exhibit a clear chain relationship: exercise identity first enhances emotion regulation capacity, which in turn fosters autonomous motivation for physical activity, ultimately promoting higher levels of perceived social self-efficacy. The findings aim to enrich theoretical understanding of identity-based health behavior and provide practical implications for designing interventions that enhance both emotional resilience and social competence among young adults.

## 2 Materials and methods

### 2.1 Procedure and participants

This study utilized a random cluster sampling method, selecting four public regular higher education institutions from each of the eastern, central, and western regions of Shandong Province, China, encompassing a total of 16 prefecture-level administrative divisions. From each participating institution, 90 students were recruited, resulting in the distribution of 1,080 questionnaires. All respondents shared the same ethnic background and comparable socioeconomic status. After excluding invalid responses–such as those with patterned answers, incomplete entries, or other irregularities-−1,029 valid questionnaires were retained, yielding a valid response rate of 95.3%. Of the valid responses, 594 (57.7%) were from female students and 435 (42.3%) from male students, with a mean age of 18.9 years (SD = 0.80). A preliminary differential analysis revealed no statistically significant differences across institutions, supporting the use of the full dataset for subsequent analysis.

Prior to data collection, informed consent was obtained from school administrators, homeroom teachers, and individual participants. The survey was administered in a group setting, following principles of voluntary involvement, data confidentiality, and respondent anonymity. Control variables such as age and gender were included in the questionnaire. Data were collected between September 10 and 30, 2025, with participants allotted 10 mins to complete the survey.

## 3 Measures and instruments

### 3.1 Exercise Identity Scale (EIS)

The Exercise Identity Scale (EIS), developed by Anderson and Cychosz (1994), was used in this study. The Chinese revised version by Li et al. (2019) was administered to assess exercise identity among university students. The scale consists of 9 items rated on a 5-point Likert scale, ranging from 1 (strongly disagree) to 5 (strongly agree), designed to evaluate the extent to which individuals identify as exercisers. Higher scores indicate a stronger level of exercise identity. The scale demonstrated good psychometric properties in the original validation, with a test-retest reliability of 0.79 and a Cronbach's α coefficient of 0.902 (Li et al., 2019). The results of confirmatory factor analysis indicated that the fit indices for the motivation dimensions were satisfactory: χ^2^/df = 2.197, RMSEA = 0.075, GFI = 0.909, CFI = 0.943, AGFI = 0.906, thus supporting the construct validity of the scale. Moreover, the scale demonstrated excellent reliability in this study, with a Cronbach's α coefficient of 0.938.

### 3.2 The scale of Perceived Social Self-Efficacy (PSSE)

This study employed the Perceived Social Self-Efficacy Scale developed by Smith and Betz (2000). The Chinese version, revised by Meng et al. (2007), was used. The scale comprises 18 items designed to measure an individual's confidence and belief in their ability to engage in social activities and maintain interpersonal relationships. Responses are recorded on a 5-point Likert scale, ranging from “no confidence at all” to “complete confidence.” Higher total scores indicate a higher level of perceived social self-efficacy. The results of confirmatory factor analysis revealed the following fit indices: χ^2^/df = 3.12, RMSEA = 0.046, GFI=0.909, CFI = 0.953, AGFI = 0.941, confirming that the multidimensional structure of the scale was well-supported. Furthermore, the scale exhibited high internal consistency, with a Cronbach's alpha coefficient of 0.912.

### 3.3 Difficulties in Emotion Regulation Scale (DERS-16)

This study utilized the brief version of the Difficulties in Emotion Regulation Scale (DERS-16) developed by Bjureberg et al. (2016). The scale consists of 16 items across five dimensions: Lack of Emotional Clarity (2 items), Difficulties Engaging in Goal-Directed Behavior (3 items), Impulse Control Difficulties (3 items), Limited Access to Effective Emotion Regulation Strategies (5 items), and Non-acceptance of Emotional Responses (3 items). Responses are rated on a 5-point Likert scale ranging from 1 (Almost Always) to 5 (Almost never). The Chinese version of the DERS-16 was adapted by Wang et al. (2021), who reported a Cronbach's α of 0.91 and test–retest reliability coefficients of 0.85, 0.83, 0.80, 0.88, and 0.81 for the five subscales, respectively, indicating good reliability and stability among Chinese university students. The results of confirmatory factor analysis demonstrated that χ^2^/df = 3.35, RMSEA = 0.049, CFI = 0.937, GFI = 0.926, AGFI = 0.913, confirming that the multidimensional structure of the scale was well-supported. Additionally, the internal consistency reliability coefficient of the scale in this study reached 0.903.

### 3.4 Motivation for Physical Activity Measure-Revised (MPAM-R)

The Chinese version of the Motivation for Physical Activity Measure-Revised (MPAM-R), revised by Chen et al. (2013), was used to measure the intrinsic motivation levels of university students. This scale is an adapted and simplified version of the original instrument developed by Ryan et al. (1997). It consists of 15 items across five subscales: enjoyment motivation, competence motivation, appearance motivation, fitness motivation, and social motivation. Responses are rated on a 5-point Likert scale ranging from 1 (not at all) to 5 (very strongly), with higher scores indicating stronger levels of intrinsic motivation. The original Chinese version demonstrated good internal consistency, with a Cronbach's α coefficient of 0.908 (Chen et al., 2013). The results of confirmatory factor analysis indicated that the fit indices for the motivation dimensions were satisfactory: χ^2^/df = 2.98, RMSEA = 0.044, CFI = 0.963, GFI = 0.934, AGFI = 0.913, thus supporting the construct validity of the scale in this study. Moreover, the scale demonstrated excellent reliability, with a Cronbach's α coefficient of 0.939.

### 3.5 Research procedure and statistical analysis

(1) Data analysis was performed using IBM SPSS Statistics 26.0. Descriptive statistics and correlation analyses were conducted for key variables, including exercise identity, perceived social self-efficacy, emotion regulation, and motivation for physical activity. Additionally, common method bias was assessed.

(2) The mediation analysis was carried out using Model 6 from the SPSS macro PROCESS (Hayes, 2013). This analysis evaluated: (a) the direct effect of exercise identity on perceived social self-efficacy; (b) the mediating roles of emotion regulation and motivation for physical activity; and (c) the chain mediating effect involving emotion regulation and motivation for physical activity.

## 4 Results

### 4.1 Common method deviation test

This study employed a questionnaire-based survey approach. To address potential common method bias, several procedural and statistical remedies were implemented. At the questionnaire design stage, reverse-scored items were incorporated into the Difficulties in Emotion Regulation Scale. During data collection, on-site administration was adopted, which included real-time assistance and immediate retrieval of completed questionnaires to enhance data accuracy. Furthermore, Harman's single-factor test was conducted to statistically assess common method variance. An unrotated exploratory factor analysis including all questionnaire items yielded eight factors with eigenvalues greater than 1, with the largest factor accounting for 34.71% of the variance. This figure is below the 40% threshold recommended by Hair et al. (1998), suggesting that common method bias is not a major concern in this study.

### 4.2 Descriptive statistical and correlation analysis

Table 1 presents the results of Pearson correlation analyses conducted to examine bivariate relationships among the study variables. This analysis served two primary purposes: first, to provide a preliminary test of the hypothesized directional relationships between exercise identity, perceived social self-efficacy, emotion regulation, and motivation for physical activity; second, to identify potential confounding variables (e.g., age, gender) by assessing their associations with key study variables, thereby establishing a foundation for subsequent regression and mediation analyses.

**Table 1:** Descriptive statistics and correlation analysis.

**Variable**	**M ±SD**	**Gender**	**Age**	**Exercise identity**	**Perceived social self-efficacy**	**Emotion regulation**	**Motivation for physical activity**
Gender	1.58 ± 0.49	1					
Age	18.87 ± 0.79	0.066	1				
Exercise identity	3.34 ± 0.88	−0.258^**^	0.062	1			
Perceived social self-efficacy	3.58 ± 0.80	−0.128^**^	−0.009	0.594^***^	1		
Emotion regulation	4.03 ± 0.80	0.047	−0.106	0.177^***^	0.303^***^	1	
Motivation for physical activity	4.22 ± 0.63	0.050	−0.055	0.413^***^	0.556^***^	0.209^***^	1

1. Values on the diagonal represent the autocorrelation coefficient (r=1) of each variable with itself, conforming to the standard presentation specification of correlation matrices; 2. N = 1029; 3. ^**^p <0.01, ^***^p <0.001; 4. Gender coding: 1 = Male, 2 = Female.

As shown in Table 1, exercise identity, perceived social self-efficacy, emotion regulation, and motivation for physical activity were all significantly intercorrelated (p <0.001). Specifically, exercise identity demonstrated significant positive correlations with perceived social self-efficacy, emotion regulation, and motivation for physical activity. Additionally, significant gender differences were observed in exercise identity and perceived social self-efficacy (*p* < 0.01), whereas age was not significantly correlated with any of the measured variables.

Table 2 presents gender differences in Exercise Identity, Perceived Social Self-Efficacy, Emotion Regulation, and Motivation for Physical Activity. Significant gender differences were observed specifically in Exercise Identity and Perceived Social Self-Efficacy, with males scoring significantly higher than females. In contrast, no significant gender differences were found in either Emotion Regulation or Motivation for Physical Activity.

**Table 2:** Differences in gender.

**Variable**	**Gender**	**M ±SD**	***t***
Exercise identity	Female	3.14 ± 0.82	8.46^***^
	Male	3.60 ± 0.88	
Perceived social self-efficacy	Female	3.50 ± 0.77	4.14^***^
	Male	3.70 ± 0.82	
Emotion regulation	Female	4.07 ± 0.76	−1.63
	Male	3.98 ± 0.85	
Motivation for physical activity	Female	4.25 ± 0.59	−1.55
	Male	4.19 ± 0.69	

N = 1029. ^***^P <0.001.

### 4.3 Significance test of mediation effect

In this study, the mediation effect was tested using Model 6 of the SPSS macro PROCESS developed by Hayes (2013), with 5,000 bootstrap samples. The regression results are presented in Table 3. Perceived Social Self-Efficacy was specified as the dependent variable, Emotion Regulation and Motivation for Physical Activity as mediators, and age and gender as control variables.

**Table 3:** Regression analysis of the relationship between variables.

**Variable**	**Model 1 perceived social self-efficacy**		**Model 2 emotion regulation**		**Model 3 motivation for physical activity**		**Model 4 perceived social self-efficacy**	
	β	***t***	β	***t***	β	***t***	β	***t***
Gender	−0.008	−0.174	0.151	2.971^**^	0.195	5.300^***^	−0.144	−3.561^**^
Age	−0.003	−0.081	−0.024	−1.687	−0.009	−0.311	0.013	0.398
Exercise identity	0.443	17.273^***^	0.319	6.606^***^	0.315	14.900^***^	0.236	9.358^***^
Emotion Regulation	-	-	-	-	0.091	4.006^***^	0.175	7.087^***^
Motivation for Physical Activity	-	-	-	-	-	-	0.525	15.518^***^
R^2^	0.240		0.058		0.219		0.423	
F	80.900^***^		16.035^***^		57.292^***^		124.756^***^	

^**^P <0.01,^***^P <0.001.

Model 1 indicated that Exercise Identity significantly and directly predicted Perceived Social Self-Efficacy [β = 0.443, 95% CI (0.393, 0.494), *p* < 0.001]. Model 2 showed that Exercise Identity positively predicted Emotion Regulation [β = 0.319, 95% CI (0.133, 0.245), *p* < 0.001]. Model 3 demonstrated that Exercise Identity positively predicted Motivation for Physical Activity [β = 0.332, 95% CI (0.274, 0.366), *p* < 0.001], and Emotion Regulation also had a significant direct and positive effect on Motivation for Physical Activity [β = 0.091, 95% CI (0.046, 0.135), *p* < 0.001]. Model 4 revealed that when Exercise Identity, Emotion Regulation, and Motivation for Physical Activity were included simultaneously, all three significantly and positively predicted Perceived Social Self-Efficacy [β = 0.236, 95% CI (0.187, 0.286), *p* < 0.001]; [β = 0.175, 95% CI (0.126, 0.223), *p* < 0.001]; [β = 0.525, 95% CI (0.458, 0.591), *p* < 0.001], respectively.

**Figure 2:**
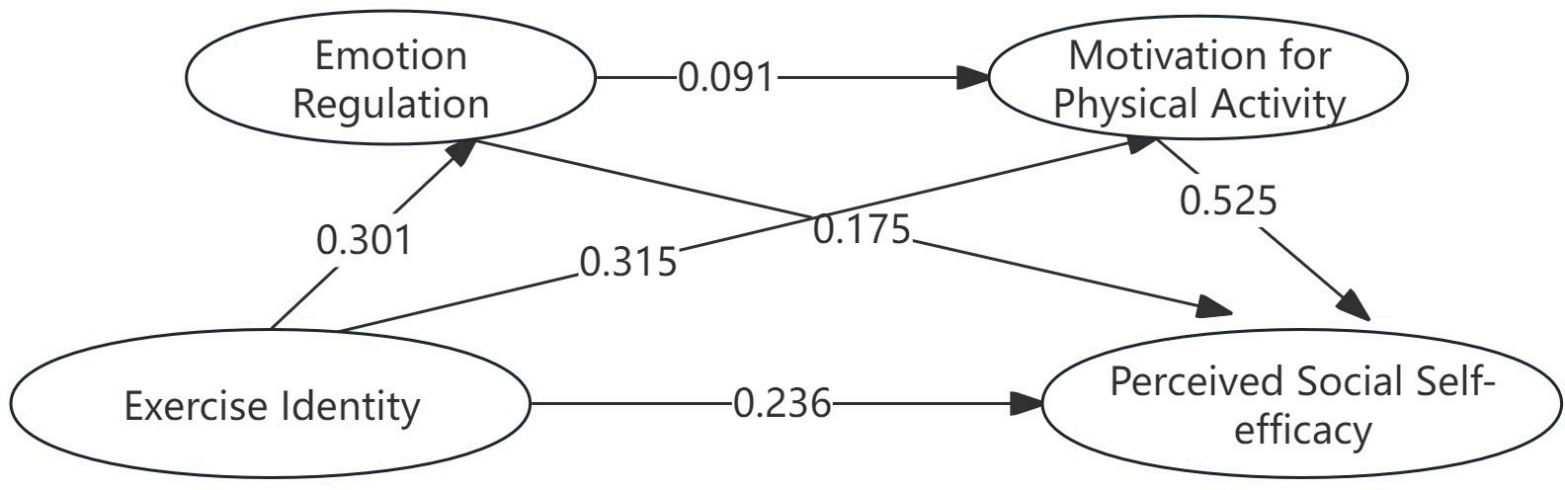
The chain-mediated mediation path of exercise identity to perceived social self-efficacy correlated with any of the measured variables.

The results of the chain mediation analysis are presented in Table 4 and Figure 2. The analysis revealed a significant total indirect effect of 0.207 [95% CI = (0.171, 0.247)]. Specifically, the indirect effect through Motivation for Physical Activity alone was 0.165 [95% CI = (0.133, 0.201)], accounting for 37.246% of the total effect. The indirect effect through the sequential pathway of Emotion Regulation and then Motivation for Physical Activity was 0.009 [95% CI = (0.004, 0.016)], accounting for 14.88% of the total effect. The indirect effect through Emotion Regulation alone was 0.033 [95% CI = (0.017, 0.053)], accounting for 7.449% of the total effect. These results collectively support the proposed chain mediation model in the relationship between Exercise Identity and Perceived Social Self-Efficacy.

**Table 4:** Bootstrap analysis of significance test of intermediary effect.

**Influence path**	**Indirect Effect**	**95% confidence interval**			**Ratio of Total Effect**
		**BootSE**	**BootLLCI**	**BootULCI**	
Total Indirect Effect	0.207	0.020	0.171	0.247	46.726
Ind1	0.033	0.009	0.017	0.053	7.449
Ind2	0.165	0.017	0.133	0.201	37.246
Ind3	0.009	0.032	0.004	0.016	0.203
Ind1-Ind2:	−0.132	0.021	−0.173	−0.092	-
Ind1-Ind3:	0.024	0.009	0.010	0.043	-
Ind2-Ind3:	0.156	0.018	0.122	0.193	-

Ind1:Exercise identity → Emotion Regulation → Perceived Social Self-efficacy; Ind2:Exercise identity → Motivation for Physical Activity → Perceived Social Self-efficacy; Ind3:Exercise identity → Emotion Regulation → Motivation for Physical Activity → Perceived Social Self-efficacy. Ind1-Ind2:Compares the difference in indirect effect sizes between the Ind1 and Ind2; Ind1-Ind3: Compares the difference in indirect effect sizes between Ind1 and Ind3; Ind2-Ind3: Compares the difference in indirect effect sizes between Ind2 and Ind3. BootLLCI = Lower limit, BootULCI = Upper limit. BootSE = Standard Error.

## 5 Discussion

### 5.1 The relationship between exercise identity and perceived social self-efficacy

The present study demonstrates a significant positive predictive effect of exercise identity on college students' perceived social self-efficacy (PSSE) (β= 0.443, *p* < 0.001). This finding supports the framework of identity theory, which maintains that individuals tend to enact behaviors consistent with their core identities to preserve self-concept coherence (Stets and Carter, 2012). These findings extend the existing empirical evidence connecting physical activity to psychosocial development by shedding light on the mechanism whereby a salient exercise identity fosters social confidence. This relationship appears to be driven by three interrelated processes: behavioral practice, self-concept reinforcement, and social feedback loops. A strong exercise identity operates as a powerful intrinsic motivator for sustained engagement in physical activity (Anderson and Cychosz, 1994).

Exercise contexts are diverse, encompassing both group settings and individual activities, and both types contribute to PSSE through distinct but complementary mechanisms. Group exercise environments inherently require communication, coordination, and collaboration, providing direct opportunities for social interaction (Reifsteck et al., 2016). For individual exercisers, the process centers on self-regulation and personal mastery: consistent self-discipline, goal-setting, and self-monitoring during solo activity enhance self-control and perceived personal efficacy (Gu, 2025). These competencies generalize to social domains by boosting self-assurance in initiating and maintaining social interactions—for example, the confidence gained from overcoming physical challenges independently translates to greater comfort in navigating new social situations (Brouwer et al., 2010). Through repeated engagement in these diverse exercise experiences, individuals accumulate mastery experiences, which, according to Bandura (1997) self-efficacy theory, constitute the most reliable source of efficacy beliefs. For group exercisers, successfully coordinating with teammates, achieving shared goals, or resolving minor interpersonal challenges fosters a sense of competence in managing social dynamics (Ma et al., 2023). For individual exercisers, overcoming fatigue, adhering to self-imposed schedules, or progressing toward personal fitness goals builds resilience and self-trust, which extend to social interactions by reducing fear of rejection or failure (Haslam et al., 2016). The deep internalization of an “exerciser” role—whether shaped by group or individual activity—fosters a stable self-schema that integrates positive self-cognitions regarding one's competence, discipline, and adaptability (Strachan et al., 2025). This salient exercise identity further predicts adaptive attributions in socially challenging situations, and this process interacts with individuals' pre-existing mindsets. For those already possessing a growth mindset, exercise identity reinforces and amplifies proactive attribution patterns: they naturally link social successes to effort and strategy, and reframe setbacks as opportunities for learning (Ntoumanis et al., 2018). For individuals with a more fixed mindset, a strong exercise identity acts as a catalyst for mindset shift—repeated experiences of improving physical skills through practice (in both group and individual settings) demonstrate that abilities are malleable, encouraging them to adopt proactive attributions in social contexts (Cui and Zhou, 2025). This identity-guided cognitive pattern, whether reinforcing or transforming existing mindsets, enables students to approach social difficulties with confidence rather than avoidance, thereby reinforcing their PSSE.

Notably, exercise identity—even when developed through individual activities—is not formed in isolation. Individual exercisers often engage with broader social networks related to their activity, such as online communities, fitness trackers with social sharing features, or casual interactions with fellow exercisers (e.g., greeting runners during a jog, joining virtual challenge groups) (Uygurta and Alar, 2023). These interactions provide external validation of the exerciser role: peers, family, or even strangers may recognize and affirm their commitment to an active lifestyle, strengthening their sense of belonging (Luo et al., 2025). Additionally, the social approval of an active lifestyle in broader cultural contexts reinforces identity salience—individuals who exercise independently may still experience social benefits such as increased approachability or shared identity with other active individuals in casual social encounters (Haslam et al., 2016). Even without direct group participation, the exerciser identity connects individuals to a larger “community of active people,” fostering social confidence through indirect social validation.

Exercise identity is thus embedded within a flexible network of behavioral and social processes, whether shaped by group or individual activity. Regular participation in physical activity—regardless of setting—facilitates repeated experiences of mastery, self-regulation, and (direct or indirect) social feedback. Recognition and affirmation from others (in group settings) or from broader social contexts (for individual exercisers) serve as powerful mechanisms that consolidate both exercise identity and perceived social competence. These reciprocal interactions create a positive feedback cycle: identity validation boosts confidence, which subsequently promotes deeper social engagement (for group exercisers) or greater comfort in social interactions (for individual exercisers), further reinforcing the identity.

Together, these findings position exercise identity as a key psychosocial resource that fosters PSSE among college students, regardless of whether their exercise is group-based or individual. By supporting adaptive self-concept regulation, building self-efficacy through mastery experiences, and facilitating (direct or indirect) social feedback, a strong exercise identity establishes a self-sustaining ecosystem conducive to social confidence. For emerging adults navigating the challenges of identity formation and social adaptation, cultivating a positive exercise identity may thus represent a viable and sustainable pathway toward enhanced social adaptability and psychological resilience.

### 5.2 The mediating role of emotion regulation

The findings of the present study indicate that emotion regulation partially mediates the relationship between exercise identity and perceived social self-efficacy (PSSE), suggesting that the beneficial influence of a strong exercise identity on social confidence operates, in part, through the enhancement of emotional management abilities. This mechanism reflects a broader psychological process in which self-identity shapes affective functioning, which in turn supports adaptive social behaviors.

Emotion regulation refers to the ability to modulate emotional responses to achieve goal-directed behavior (Phillips and Power, 2007). Individuals with a salient exercise identity are more likely to engage in regular and structured physical activity, which serves as a natural context for developing emotional regulation skills. Empirical evidence demonstrates that consistent exercise contributes to lower emotional reactivity, enhanced mood stability, and greater psychological resilience (Gao et al., 2025). From a self-regulatory perspective, adopting the “exerciser” identity promotes goal-oriented behavior and self-discipline, which extend beyond the physical domain into emotional regulation processes (Su et al., 2024). The routines and challenges inherent in exercise—overcoming fatigue, managing frustration, or coping with performance setbacks—provide repeated opportunities to practice emotion regulation strategies such as reappraisal, attentional control, and self-soothing. Over time, these habitual responses form an integrated part of the self-concept, reinforcing both self-control and emotional competence. Furthermore, neurobiological research suggests that regular physical activity modulates the hypothalamic–pituitary–adrenal (HPA) axis and increases endorphin and serotonin levels, thereby reducing stress sensitivity and improving affective balance (Guo and Zhang, 2022). In this sense, exercise identity not only motivates behavior but also contributes to emotion regulation capacity at both the cognitive and physiological levels.

Effective emotion regulation is fundamental to social functioning and interpersonal success. Individuals who can identify, interpret, and manage their emotions appropriately are better equipped to navigate social stressors, resolve conflicts, and express empathy (Di Maggio et al., 2016). For college students, the ability to regulate emotions during social interactions such as managing embarrassment in new peer groups or frustration during collaborative projects significantly influences their perceived social competence. Empirical studies have consistently shown that emotion regulation strategies like cognitive reappraisal and positive refocusing predict higher levels of social efficacy and lower social anxiety (Gross and John, 2003). Conversely, reliance on maladaptive strategies, such as suppression or rumination, is associated with impaired social confidence and heightened interpersonal tension (Aldao et al., 2010). In this regard, emotion regulation functions as a psychological bridge between personal identity and social competence: it translates self-concept into adaptive behavior through affective control.

Notably, the transfer of emotion regulation skills from exercise contexts to social settings is not universal. Individuals with a well-established exercise identity may be more likely to employ regulatory strategies, such as deep breathing, cognitive reframing, or focusing on controllable factors, which are routinely practiced during physical activity, when confronted with socially demanding situations like public speaking or group decision-making (Reifsteck et al., 2016), a salient exercise identity is closely linked to enhanced self-regulatory capacities, as the consistent pursuit of exercise-related goals fosters habitual use of adaptive emotion regulation strategies (Strachan et al., 2025). However, this tendency can be moderated by individual differences. For example, individuals with lower trait self-efficacy or higher social anxiety often struggle to generalize these skills, as situational pressures in social interactions, including fear of judgment, can override the regulatory competencies developed through exercise (Na et al., 2024). Additionally, the effectiveness of skill transfer may depend on the similarity between exercise and social contexts: structured, low-pressure exercise environments (e.g., individual fitness training) may provide fewer opportunities to practice social-specific emotion regulation than group-based sports or team activities, limiting the generalizability of learned strategies. Moreover, the effectiveness of skill transfer likely depends on contextual similarity. Structured, low-pressure exercise environments, such as individual fitness training, may offer fewer opportunities to practice socially relevant emotion regulation compared to group-based sports or team activities, thereby limiting the generalizability of learned strategies. Despite these moderating factors, the overall pattern indicates that a strong exercise identity tends to foster emotion regulation capacities that frequently extend into social domains, with individual differences and contextual features shaping the strength of this transfer. Successful transfer of these self-regulatory skills, in turn, enhances perceived social efficacy and confidence.

The mediating effect observed in this study underscores that emotion regulation is a key pathway through which exercise identity exerts its social benefits. The mechanism can be conceptualized as a two-step process: (1) Identity-driven emotional regulation: A salient exercise identity promotes consistent engagement in emotionally beneficial behaviors, reinforcing adaptive emotion regulation strategies. (2) Emotion-driven social efficacy: Enhanced emotional regulation reduces anxiety and fosters composure and authenticity during social interactions, thereby elevating perceived social self-efficacy. This model aligns with emerging research demonstrating that emotional regulation capacities mediate the link between health-promoting identities (e.g., exerciser, volunteer, or leader) and psychosocial outcomes such as resilience, self-esteem, and social competence (Tamir, 2016; Yan and Lu, 2025).

These results suggest that emotion regulation plays a pivotal intermediary role in the pathway from exercise identity to PSSE. Exercise identity cultivates a structured behavioral context and mindset conducive to emotional mastery, which in turn enhances social confidence. This mediational pattern highlights the affective dimension of identity-based motivation, offering new insight into how identity-oriented interventions can be leveraged to strengthen both emotional and social functioning among university students.

### 5.3 The mediating role of motivation for physical activity

The present study provides evidence that motivation for physical activity serves as an important mediating mechanism linking exercise identity to perceived social self-efficacy (PSSE). This finding aligns with Self-Determination Theory (Deci and Ryan, 2000), which posits that the quality of motivation, specifically its type and source, determines behavioral persistence and psychological outcomes. Individuals who strongly internalize an “exerciser” identity are more likely to exhibit autonomous and integrated forms of motivation, which are fueled by inherent satisfaction, a sense of competence, and social connection rather than by external pressures or obligations. This internalized motivation not only sustains engagement in physical activity but also fosters the psychological and social benefits that accompany such behavior (Yan et al., 2020).

A salient exercise identity fosters congruence between self-concept and behavior. When individuals perceive exercise as a central aspect of who they are, they are more likely to pursue physical activity for intrinsic and self-endorsed reasons, such as enjoyment and personal growth (Hagger and Chatzisarantis, 2011). Those with stronger exercise identity report higher scores on subscales of enjoyment, competence, and social interaction (Teixeira et al., 2012). Autonomous motivation enhances key outcomes such as persistence, satisfaction, and vitality, all of which are closely tied to self-efficacy and wellbeing. Critically, the process of motivation internalization is self-perpetuating. According to Self-Determination Theory, when the basic psychological needs for autonomy, competence, and relatedness are satisfied, they facilitate more self-determined motivation, which in turn consolidates one's identity and sense of personal agency (Ryan and Deci, 2014). This creates an upward spiral in which exercise identity and autonomous motivation reciprocally strengthen one another, thereby promoting adaptive functioning across both physical and social domains.

Physical activity motivation generates repeated opportunities for social interaction, cooperative behavior, and evaluative feedback, particularly when it is oriented toward social and competence-related goals. Group-based exercise environments, which rely on coordination and mutual support, enable participants to develop social mastery and experience inclusion (Ntoumanis et al., 2021), thereby directly strengthening social self-efficacy through successful collaborative engagement. Intrinsic motivation further supports this process by fostering positive affect, vitality, and self-esteem, which indirectly facilitate social functioning (Standage et al., 2012). Individuals who find exercise enjoyable and volitional tend to exhibit greater openness and confidence in social contexts. Conversely, those motivated by external controls often experience diminished enjoyment and social disengagement, which compromises the development of social self-efficacy (Teixeira et al., 2012). Therefore, the regulatory dynamics of motivation determine whether physical activity promotes social growth or remains a neutral routine. This underscores that the relationship between exercise identity and PSSE is mediated not only cognitively but also motivationally and affectively. Exercise identity establishes a coherent self-concept, while autonomous motivation channels this identity into sustained behavioral engagement and affectively rewarding experiences that build social confidence. Ultimately, autonomous motivation serves as the core mechanism translating exercise identity into social self-efficacy.

### 5.4 The chain mediating effect of emotion regulation and motivation for physical activity

The present study further reveals that emotion regulation and motivation for physical activity jointly exert a significant chain mediating effect in the relationship between exercise identity and perceived social self-efficacy (PSSE). This finding suggests that the pathway from identity to social confidence unfolds through a sequential psychological mechanism: exercise identity enhances individuals' emotional regulation abilities, which subsequently promote self-determined motivation for physical activity, ultimately leading to greater perceived social self-efficacy. This integrated process highlights the dynamic interplay between affective regulation and motivational activation within identity-based behavior.

A strong exercise identity cultivates consistent engagement in structured physical activities that inherently demand emotional control—overcoming fatigue, frustration, and self-doubt. Such repeated experiences facilitate the acquisition and internalization of emotion regulation skills, including cognitive reappraisal and attentional redirection (Gross, 1998). Empirical research has demonstrated that individuals who view themselves as “exercisers” exhibit greater emotional resilience and lower stress reactivity (Mikkelsen et al., 2017). This affective stability forms the foundation upon which motivation quality is built: individuals capable of regulating negative affect are more likely to maintain autonomous motivation, perceiving exercise as a source of pleasure and self-expression rather than as an obligation (Tamir, 2016).

Effective emotional management fosters a sense of autonomy and mastery. According to Deci and Ryan (2000), these two factors are central components of self-determined motivation. When individuals can down-regulate anxiety or frustration associated with physical exertion, they are more likely to internalize positive meanings of exercise, such as enjoyment, competence, and social connection (Ntoumanis et al., 2021). Conversely, those who lack emotional control may experience exercise as aversive, leading to externally regulated or amotivated participation. Hence, emotion regulation acts as a psychological catalyst, transforming transient behavioral engagement into sustained, self-driven participation in physical activity (Zhang, 2024).

The influence of autonomous motivation transcends physical behavior to affect multiple psychosocial domains. By fostering positive affect, vitality, and self-esteem, self-determined motivation enhances individual's interpersonal confidence and social openness (Standage et al., 2012). In group exercise settings, which inherently require communication and collaboration, individuals learn to transfer these socio-affective resources into concrete social self-efficacy. Through this mechanism, emotion regulation indirectly supports social confidence—not by directly modifying behavior, but by improving the quality of motivation that underlies sustained engagement in socially interactive exercise.

The chain mediation model validated in this study, Exercise Identity → Emotion Regulation → Motivation for Physical Activity → Perceived Social Self-Efficacy, offers a nuanced understanding of how self-concept in the exercise domain cascades through affective and motivational processes to shape social outcomes. This sequential pathway demonstrates that emotional control acts as the psychological substrate for internalized motivation, which in turn empowers effective social functioning. Theoretically, this model integrates Identity Theory and Self-Determination Theory, suggesting that identity-consistent behavior is not sustained solely through cognitive alignment but through emotionally regulated motivation. Practically, interventions that combine emotion regulation training (e.g., mindfulness, reappraisal exercises) with autonomy-supportive physical activity programs may yield synergistic benefits for students' emotional wellbeing and social adaptability.

## 6 Limitations and future prospects

Although the present study provides valuable insights, several limitations warrant consideration. First, the cross-sectional design restricts causal inference; future research should employ longitudinal or experimental approaches to examine the dynamic evolution of exercise identity and PSSE over time. Second, cultural factors may shape how identity and motivation function-replication in diverse cultural contexts will enhance generalizability. Third, incorporating behavioral and physiological measures (e.g., heart rate variability, social network density) could complement self-reported data and clarify the underlying mechanisms. Future studies may also explore additional mediators-such as self-compassion, group belongingness, or emotional intelligence-to further elaborate how exercise-related identities influence psychosocial wellbeing.

## 7 Conclusion

The study found that exercise identity significantly and positively predicts college students' perceived social self-efficacy. This relationship is partially mediated by emotion regulation and motivation for physical activity, which also form a sequential chain mediation pathway. Specifically, students with a stronger exercise identity show better emotional regulation abilities and higher autonomous motivation, which together enhance their confidence in social interactions. These results highlight that developing a positive exercise identity can effectively promote college students' emotional stability, social adaptability, and overall psychological wellbeing.

## Data Availability

The datasets presented in this study can be found in online repositories. The names of the repository/repositories and accession number(s) can be found in the article/supplementary material.
